# The Cariostatic Mechanisms of Fluoride—An Updated Review

**DOI:** 10.3390/dj14070390

**Published:** 2026-06-24

**Authors:** Ivana Šutej, Krešimir Bašić, Kristina Peroš

**Affiliations:** School of Dental Medicine, University of Zagreb, 10000 Zagreb, Croatia; isutej@sfzg.hr (I.Š.); peros@sfzg.hr (K.P.)

**Keywords:** fluoride, cariostatic mechanisms, remineralization

## Abstract

Fluoride remains the keystone of evidence-based caries prevention by stabilizing the mineral balance at the tooth–biofilm–saliva interface. Contemporary understanding emphasizes a predominantly post-eruptive, topical mode of action where fluoride inhibits demineralization and accelerates remineralization. This interfacial catalysis is reinforced by pH-responsive calcium-fluoride-like reservoirs that release fluoride during acid challenges. While community water fluoridation confers population-level reductions, the most effective approach is sustaining low-level fluoride in the biofilm environment. Evidence confirms that toothpastes with 1000–1500 ppm fluoride provide a dose–response benefit in children, while 5000 ppm concentrations are indicated for high-risk scenarios such as root caries and xerostomia. Beyond physicochemical effects, fluoride modulates the oral microbiome by inhibiting bacterial enzymes and proton pumps, shifting community function toward a health-associated state without reducing overall diversity. In restorative dentistry, glass ionomer cements offer superior preventive effects against secondary caries compared to amalgam; however, marginal integrity, adhesive performance, and clinical technique, rather than fluoride release alone, remain the primary determinants of success. Despite well-known risks associated with high systemic intake, such as fluorosis, current evidence does not indicate genotoxic or adverse microbiome effects in humans from routine topical use of standard fluoride products at recommended preventive concentrations. Overall, fluoride’s cariostatic value rests on frequent, low-level exposures that maintain tissues in a repair-favoring state.

## 1. Introduction

Fluoride remains the keystone of evidence-based caries prevention because it stabilizes the mineral balance at the tooth–biofilm–saliva interface under real-world, diet-modulated conditions. Contemporary understanding emphasizes a predominantly post-eruptive, topical mode of action. Trace fluoride in plaque fluid adsorbs to carbonated apatite, lowers the activity product required for precipitation, and thereby inhibits demineralization during acid challenges while accelerating remineralization as pH recovers. This interfacial catalysis is reinforced by pH-responsive calcium-fluoride-like reservoirs that dissolve when the biofilm acidifies, releasing fluoride where and when it is most needed [1,2,3]. Accordingly, the most effective approach is to sustain low-level fluoride in the biofilm environment rather than depend on infrequent peaks [1,2]. Community water fluoridation confers population-level caries reductions, but the magnitude is modest and context-dependent. In modern contexts with widespread toothpaste use, the effect size is smaller [4]. Communications accompanying the same update stress interpreting fluoridation within today’s exposure landscape and pairing it with measures that address behavioral risks and access to care [4]. In practical terms, fluoridation functions best as supportive baseline infrastructure, while the emphasis remains on sustaining regular, low-level topical availability across the day. This aligns with thermodynamic expectations that, as topical exposure rises, the added value of systemic fluoride diminishes [4]. This diminishing marginal value should not, however, be equated with redundancy. Community water fluoridation continues to provide caries-preventive benefit at the population level, and its principal contemporary strength lies in equity: it reaches individuals irrespective of health literacy, motivation, or access to dental products and professional care, thereby reducing disparities that targeted topical measures may not address. Its role is therefore best understood as complementary to, rather than superseded by, widespread topical fluoride use, with the relative contribution of each varying across populations and exposure settings. For home care, the key trial evidence is the Cochrane review showing that toothpastes with ≥1000–1500 ppm fluoride prevent caries better than non-fluoride pastes and display a dose–response in children and adolescents. These findings still anchor policy and guidance [5]. Guideline bodies translate this into twice-daily brushing with age-appropriate fluoride toothpaste plus risk-based professional adjuncts, including specific toddler dosing to balance efficacy and fluorosis risk, an approach reinforced by recent pediatric best-practice statements [6,7,8]. Observational data indicate many caregivers still overuse infant toothpaste despite “grain-of-rice” advice, highlighting the need for precise, hands-on instruction [9]. In parallel, updated syntheses on topical agents refine fluorosis risk attribution to modern products, helping calibrate timing and quantity of use in young children [10].

Mechanistically, three consensus points are relevant to clinical translation. First, fluoride lowers the effective critical pH for enamel and dentin, so after sugar exposures, the tissues spend more of the cycle in a repair-favoring state [1,2]. Second, caries control is fundamentally about maximizing time in supersaturation at the interface where behaviors and care pathways that increase the frequency of low-level fluoride in plaque and saliva produce the greatest returns [1,2,4,5,7,8]. Third, although antimicrobial actions such as enolase and H^+^-ATPase inhibition occur, their in vivo contribution is secondary to the physicochemical effects on mineral balance at the tissue surface. Consequently, the most visible clinical gains are seen in non-cavitated lesions through surface rehardening and slowed lesion progression [1,2].

This article is a narrative review. It does not follow a formal systematic review protocol; rather, it synthesizes mechanistic, clinical, and guideline-level evidence to provide an updated, integrative account of the cariostatic mechanisms of fluoride. Relevant literature was identified through searches of PubMed/MEDLINE and the Cochrane Library, conducted between November 2025 and March 2026 and supplemented by hand-searching of reference lists and authoritative guideline documents. Key terms included combinations of ‘fluoride’, ‘caries’, ‘demineralization’, ‘remineralization’, ‘calcium fluoride reservoir’, ‘fluorapatite’, ‘enamel fluoride uptake’, ‘fluoride toothpaste’, ‘varnish’, ‘glass ionomer’, ‘secondary caries’, ‘oral microbiome’, ‘cytotoxicity’, and ‘genotoxicity’. Evidence was synthesized narratively and, where relevant, interpreted by study level (in vitro, animal, clinical, and population-based) and by exposure domain (topical versus systemic), without formal quantitative pooling.

This article is a revision and update of our previous review, adapted to the contemporary context of fluoride exposure [11]. In addition to consolidating the established interfacial model of cariostasis, including adsorbed fluoride and CaF_2_-like reservoirs, the present version substantially expands the evidence base in areas that were limited or unavailable in 2013, including nanoscale characterization of ultra-shallow enamel fluoridation and its implications for enamel solubility, contemporary clinical evidence on fluoride concentrations and indications across over-the-counter and prescription dentifrices, and updated outcomes for fluoride-releasing restorative materials in which marginal integrity and clinical technique are treated as primary determinants of secondary caries. The update also strengthens the biological sections by integrating multi-omics evidence on biofilm function and by restructuring safety and limitations according to exposure domain, distinguishing routine topical use from high-dose topical experimental exposure and from chronic high systemic intake, while future directions incorporate emerging diagnostic and implementation challenges across the life course.

## 2. Biodynamics of Caries Development

Dental caries emerges from a dynamic imbalance between physicochemical demineralization and biologically mediated remineralization at the tooth–biofilm–saliva interface. Rather than a linear event, the process fluctuates over minutes to hours in response to diet, salivary function, and biofilm metabolism. After intake of fermentable carbohydrates, plaque organisms rapidly ferment sugars, generating organic acids and a characteristic fall in biofilm pH (Stephan curve). When pH drops below the tissue-specific “critical” range, approximately 5.5 for enamel and around 6.2–6.4 for dentin/root, the plaque fluid becomes undersaturated with respect to apatite, and mineral dissolves. As pH recovers, supersaturation is restored and mineral can redeposit [12,13,14].

Saliva governs much of this physiology. Its flow rate dictates clearance of acids and sugars and delivers buffering capacity primarily via bicarbonate, supplemented by phosphate and proteins. During stimulated flow, bicarbonate levels rise steeply, elevating buffer capacity and hastening pH recovery; during unstimulated flow, reduced bicarbonate prolongs the time biofilm pH remains below critical values, favoring net mineral loss [15,16,17]. Diurnal studies confirm lower intraoral pH during sleep and more frequent excursions below critical thresholds during prolonged fasting or hyposalivation [18].

Enamel and dentin respond differently to these fluctuations. Enamel, highly mineralized and low in protein, typically develops a subsurface lesion. It has a relatively intact surface zone which overlays a more demineralized “body” created by diffusion gradients of acids in and mineral ions out. Classic histopathology reveals four zones: translucent, dark, body of lesion, and surface zone. These differences are reflecting gradients in porosity and mineral content. Kinetic constraints on ion diffusion and reprecipitation explain why the surface zone persists even as the subsurface demineralizes [19,20,21]. Dentin, with lower mineral content and a permeable collagen matrix, demineralizes at higher pH and progresses more rapidly once exposed, as acids diffuse along tubules and organic degradation follows [14].

The pellicle and salivary proteins modulate mineral thermodynamics at the crystal surface. Early-adsorbing phosphoproteins, such as statherin and acidic proline-rich proteins (aPRPs), bind to hydroxyapatite, help maintain calcium–phosphate supersaturation, inhibit spontaneous precipitation, and thus regulate both dissolution and regrowth at the enamel interface. Observational and experimental studies associate higher levels or functional activity of these proteins with more favorable mineral homeostasis [22,23].

Biofilm ecology contributes additional pH control beyond acids. Endogenous alkali generation via the urease pathway (urea → ammonia + carbonic acid) and the arginine deiminase system (ADS) can neutralize acid, shorten the time below critical pH, and select for less aciduric communities. Cross-sectional and mechanistic studies link higher plaque urease/ADS activity with lower caries experience, underscoring that caries risk reflects the balance of acid and alkali fluxes within the biofilm [24,25,26].

At the microenvironmental level, diffusion is rate-limiting. Unionized acids like lactic acid, penetrate enamel more readily than charged species, establishing steep concentration gradients. Simultaneously, dissolved calcium and phosphate diffuse outward from the lesion body. The net direction of mineral movement at any moment depends on the ion activity product in plaque fluid, itself set by pH, salivary composition, and pellicle binding. Experimental models of artificial lesions and in vivo observations show that frequent, closely spaced acid challenges compress recovery periods, shifting the area under the pH–time curve toward demineralization, whereas longer intervals between challenges permit surface rehardening and lesion arrest [13,19].

Clinically, this physiology yields several corollaries without invoking exogenous remineralizing agents. Time dependence, the cumulative time biofilm pH spends below the tissue-specific critical threshold predicts net mineral loss. Site specificity, stagnation areas with thicker biofilm and limited salivary access experience deeper pH drops and slower recovery. Host modulation, salivary flow and buffer capacity strongly influence the balance. And ecological feedback, acidogenic/aciduric selection under frequent sugar exposure reinforces further acid production, while alkali pathways and proteinaceous pellicle components support a more neutral milieu [12,13,16,24].

Taken together, caries development is best understood as a physiological oscillation of mineral saturation states governed by biofilm metabolism, salivary chemistry and flow, pellicle–crystal interactions, and tissue ultrastructure. Interventions that restore favorable timing, optimize natural salivary function, and maintain pellicle integrity act, by purely physiological means, to re-establish the predominance of remineralization over demineralization.

## 3. Effects of Fluoride on Enamel Solubility

Enamel solubility in cariogenic conditions reflects the thermodynamic stability of its carbonated hydroxyapatite (HAP) crystallites and the interfacial chemistry of the pellicle–biofilm–fluid phase. Fluoride modifies both. Classically, complete substitution of hydroxyls by fluoride yields fluorapatite (FAP), whose lattice is more compact and less soluble than HAP. The associated saturation state shifts the effective critical pH downward, decreasing mineral dissolution during an acid challenge [27]. Yet, contemporary evidence indicates that in vivo protection arises largely from surface-confined fluoride, adsorbed ions and ultra-shallow fluoridation of crystal surfaces, rather than wholesale conversion of enamel to stoichiometric FAP [27].

At the lattice level, fluoride substitution contracts the apatite a-axis and increases crystallinity, lowering the ion activity needed for precipitation and raising resistance to acid-driven dissolution [1,27]. Nanoscale imaging shows heterogeneous, stratified fluoride uptake in enamel, forming ultrathin, partially fluoridated hydroxyapatite shells that are less soluble than native hydroxyapatite despite being far from stoichiometric fluoroapatite [28]. In model systems, minimum solubility occurs at modest lattice fluoride (~2–3 at % F) and then plateaus, consistent with the very low fluoride levels typically measured in erupted enamel [28]. Taken together, bulk fluoroapatite is uncommon in mature crowns, yet small amounts of lattice fluoride still meaningfully slow dissolution [1,27,28].

Beyond lattice effects, interfacial fluoride drives the real-time response to an acid challenge. At sub-ppm levels in plaque fluid, it adsorbs to crystal steps/kinks, slows calcium–phosphate detachment during undersaturation, and accelerates reprecipitation as pH recovers [27]. After topical exposure, transient CaF_2_-like deposits on pellicle and in superficial porosities act as pH-responsive sources. They are stable near neutrality; they dissolve as pH falls and raise local fluoride activity exactly when dissolution pressure is highest. These reservoirs are short-lived (hours to days) and are replenished by routine use; therefore, frequency of exposure matters more than peak dose for keeping enamel less soluble during dietary acid cycles [1,29]. These mechanisms produce measurable effects. In pH-stat and microhardness models, low-level fluoride in the demineralizing solution lowers enamel dissolution rate constants and helps maintain a mineralized surface zone even when lattice fluoride is minimal [27,30].

Two conceptual clarifications are warranted. First, the frequently cited dichotomy between topical and systemic action is mechanistically less about route of delivery and more about where fluoride resides during an acid challenge. Regardless of source, cariostatic efficacy depends on fluoride activity in the aqueous phase at the crystal interface, not on the amount irreversibly built into deep enamel [27]. Second, the protective effect should be framed kinetically as well as thermodynamically: by stabilizing the surface and supplying pH-triggered fluoride, the system spends less time below the tissue-specific critical pH for dissolution, narrowing lesion progression windows between sugar exposures [1,27].

In sum, fluoride lowers enamel solubility through a synergy of shallow lattice fluoridation and interfacial adsorption/reservoir dynamics. Partial substitution in carbonate-rich apatite reduces intrinsic solubility; adsorbed fluoride and CaF_2_-like stores suppress dissolution exactly when undersaturation arises. Contemporary nanoscale and kinetic data therefore support prevention strategies that maintain frequent, low-level fluoride availability to keep enamel in a less soluble, more repair-prone state during the recurring acid–base oscillations of the biofilm environment [1,27,28,29,30].

One clinical corollary of this interface-confined mode of action warrants note. Because protection is concentrated in a thin surface layer, a remineralized or acid-resistant enamel surface may, in principle, overlie continuing subsurface or dentinal demineralization, the phenomenon historically termed “hidden” or occult caries, classically associated with widespread fluoride exposure [31,32]. The direction of this association, however, runs counter to the historical assumption: epidemiological comparisons of fluoridated and non-fluoridated populations report a lower, not higher, proportion of clinically sound surfaces harboring dentinal radiolucency, with the prevalence of hidden caries falling as water and dentifrice fluoride became widespread [33,34]. The mechanistically relevant concern is therefore not that fluoride generates occult lesions, but that surface hardening may slow their cavitation and blunt their clinical signs, narrowing the window in which they are radiographically distinguishable from sound enamel, an issue with direct implications for detection thresholds that we revisit in the Future Directions.

## 4. Fluorapatite Formation, the Role of Enamel Pretreatment on Fluoride Incorporation

Fluoride incorporation into enamel is governed not only by the chemistry of the fluoride agent but also by the state of the surface at the moment of exposure. As stated previously, in vivo the cariostatic effect is best explained by a nanometric, partially fluoridated hydroxyapatite surface layer, augmented by adsorbed F^−^ at crystal steps, rather than by bulk conversion to stoichiometric fluorapatite. Consequently, pretreatment that alters pellicle coverage, organic content, mineral exposure, or contact time can modulate fluoride uptake and the persistence of this protective layer [1,27,35,36].

Pellicle management and prophylaxis are primary determinants of uptake. Brushing and flossing before APF produce higher enamel fluoride concentrations than rubber-cup prophylaxis, indicating that abrasive pastes and rotary procedures can leave residual films or alter surface conditioning in ways that impede ion diffusion and reduce uptake [35]. Topical fluoride applied to a freshly cleaned enamel surface does not inherently compromise adhesion, supporting the practicality of pretreating clean enamel to optimize fluoride contact without sacrificing restorative performance [37,38].

Taken together, surface conditioning that exposes mineral while minimizing smear layers favors neutral NaF and MFP systems, which rely on diffusion-controlled adsorption and shallow lattice fluoridation. In contrast, with very low-pH formulations like APF or TiF_4_, removing the organic component can attenuate formation of the protective surface complex, whereas modest etching or simple pellicle removal can enhance it [39,40,41]. Despite differences in surface fluoride, plaque-fluid fluoride at endpoint remains similar across regimens, indicating that pretreatment governs where fluoride is deposited as much as how much remains bioavailable [41,42]. Deproteinizing rinses, such as sodium hypochlorite, sometimes used in adhesive dentistry, should therefore be extrapolated to fluoride pretreatment with caution. Although NaOCl can improve bonding to compromised enamel, its effect on fluoride uptake is not well established, and excessive protein removal could plausibly diminish reactivity for certain salts [43]. Nanoscale analyses align with this framework, showing heterogeneous, stratified fluoride incorporation concentrated within the outer tens of nanometers and local minima in solubility at modest lattice F^−^ substitutions; pretreatments that increase micro-topography or reduce diffusion barriers amplify this near-surface enrichment [1,27,28]. In practical terms, pretreatment determines how efficiently applied fluoride is converted into a durable, low-solubility surface layer rather than being lost to plaque fluid.

Two clinical implications follow. First, keep it simple when possible. Thorough toothbrushing/flossing to disrupt pellicle and biofilm immediately before topical fluoride can be as good as, or better than, abrasive cup prophylaxis for promoting uptake, and it avoids paste residues that impede ion access [37]. Second, match the pretreatment to the agent. Brief etching may enhance uptake for neutral fluorides; for strongly acidic systems, retain some organic scaffold and ensure adequate contact time to achieve comparable surface fluoridation [39,40,41]. Overall, enamel pretreatment is not a trivial prelude but a determinant of interfacial fluoride chemistry. By tailoring pellicle management, surface conditioning, and contact time to the chosen agent, clinicians can maximize the formation of a thin, acid-resistant, fluoridated layer which is the true locus of cariostatic benefit, without relying on wholesale fluorapatite conversion [1,27,28,35].

## 5. Cariostatic Effect of Fluoride Added to Restorative Materials

The cariostatic premise of fluoride-containing restoratives is twofold. At the outset, however, it must be emphasized that fluoride release operates as one contributor within a broader set of determinants; marginal integrity, adhesive performance, moisture control, caries risk, and clinical technique consistently emerge as the dominant factors governing secondary caries outcomes, with fluoride availability acting as an adjunctive modifier. Firstly, the sustained release and recharge of fluoride from the material–tooth interface to dampen acidogenicity and reduce enamel/dentin solubility during pH drops, and secondary localized elevation of fluoride in plaque fluid and within marginal microgaps to slow demineralization and promote repair, thereby reducing secondary caries (SC). Early reports supported this paradigm, associating higher plaque/salivary fluoride near restorations with inhibition of mutans streptococci and lactobacilli and with slower lesion progression at margins [44,45]. Contemporary evidence is more nuanced and varies by material class and clinical context.

**Glass ionomer cements (GICs) and resin-modified GICs (RMGIs).** A recent systematic review and meta-analysis concluded that GIC restorations exhibit a superior preventive effect against SC compared with amalgam and a similar effect compared with resin composites in both primary and permanent teeth, aligning with the concept that clinically relevant fluoride flux occurs at the restoration–tooth interface without compromising outcomes relative to resin [46]. While single-center observational data suggest RMGIs may show lower SC than conventional GICs—potentially due to improved adhesion and reduced microleakage—these findings should be interpreted cautiously given design limitations and short follow-ups [47]. Mechanistically, GICs demonstrate initial burst release followed by long-term low-level diffusion and are amenable to recharge from daily fluoride exposures, supporting sustained interfacial availability over the restoration’s life [48].

**Ion-releasing/bioactive resins (giomers, compomers, “bioactive” composites).** The clinical question is whether embedding fluoride-releasing or S-PRG (surface pre-reacted glass-ionomer) fillers confers SC protection comparable to GICs while retaining resin mechanical advantages [49]. An updated systematic review of “ion-releasing restorations” (IRRs) versus conventional resin composites found no overall difference in clinical effectiveness across indications, suggesting that fluoride release alone does not guarantee superior caries outcomes when adhesive strategy and marginal integrity are well controlled [50]. Evidence specific to S-PRG–based giomers in children remains emergent; a 2025 scoping review highlighted promising bioactivity signals but emphasized heterogeneity and the need for longer randomized trials with SC endpoints [51]. For compomers and nano-filled fluoride composites, recent systematic reviews map substantial variability in fluoride release by formulation, surface coating, storage medium, and recharge regimen—factors that complicate cross-study inference and may explain inconsistent clinical effects [52].

**Secondary caries and marginal adaptation.** A 2022 systematic review comparing ion-releasing materials with conventional composites found no clear reduction in SC when marginal adaptation was equivalent—underscoring that microleakage control and adhesive performance remain primary determinants of risk, with fluoride acting as an adjunct rather than a substitute for seal [53]. Conversely, in high-risk or moisture-compromised settings (e.g., cervical/root caries, partial caries removal), the combination of chemical adhesion and fluoride release in GIC/RMGI may offer pragmatic advantages that translate into similar or better SC outcomes than resin [46].

**Short-term laboratory signals vs. clinical endpoints.** In vitro/ex vivo studies frequently demonstrate higher short-term fluoride release and reduced demineralization adjacent to fluoride-containing hybrids or self-adhesive “bioactive” composites, but these signals do not consistently extrapolate to durable SC reductions in vivo [54]. Network and pairwise meta-analyses of “bioactive” restoratives reiterate that claims of caries prevention must be anchored to clinical SC outcomes rather than fluoride release alone [55].

To conclude, the weight of recent evidence supports three practical inferences. First, GIC/RMGI remain credible ion-releasing options with SC performance at least comparable to resin composites in many settings—and, in some analyses, superior to amalgam—with the added benefits of rechargeability and chemical bonding to dentin/cementum [46,56]. Second, for resin-based ion-releasing systems (giomers/compomers/bioactive composites), formulation matters: fluoride output varies widely, and current clinical evidence does not demonstrate a consistent SC advantage over well-placed conventional composites; rigorous RCTs with standardized SC diagnostics and ≥3–5-year follow-up are still needed [55]. Third, across all classes, adhesive strategy and marginal quality remain the dominant levers for SC control; fluoride release should be viewed as an adjunctive ecological modifier at the interface, not a remedy for poor isolation or technique [56].

Future trials stratify by baseline caries risk and salivary flow, report quantitative recharge protocols and plaque-fluoride kinetics, and employ uniform SC detection criteria (ICDAS-compatible) at restoration margins. Such designs will clarify when and for whom fluoride-releasing restoratives deliver clinically meaningful cariostasis beyond excellent adhesion and biofilm control.

## 6. Cariostatic Effect of Fluoride Added to Commercial or Professional Toothpastes

Fluoride dentifrices deliver low, sustained fluoride to plaque fluid during and after brushing, lowering enamel and dentin solubility during acid challenges and accelerating remineralization as pH recovers. The strongest evidence for effectiveness comes from the Cochrane review of fluoride toothpastes across concentrations, which confirms a preventive advantage over non-fluoride pastes and a dose–response in children and adolescents indicating higher concentrations to produce larger caries reductions [5].

Mechanism of action of fluoride in both, OTC and professional pastes, includes suppression of mineral dissolution during undersaturation and acceleration of remineralization process during recovery after a pH drop. Salivary fluoride remains 100–1000-fold increased for 1–2 h after brushing, maintaining protective levels between meals when used twice daily with adequate post-brushing behavior, such as minimized rinsing, which prolongs plaque-fluid fluoride activity [57].

Active compounds of dentifrices as source of fluorides usually include sodium fluoride (NaF), sodium monofluorophosphate (MFP), or stannous fluoride (SnF_2_) with different fluoride bioavailability which affects cariostatic efficacy. Stannous fluoride provides cariostatic efficacy comparable to NaF for caries prevention and adds anti-gingivitis/anti-erosion benefits when adequately stabilized, with recent reviews suggesting SnF_2_ can outperform NaF for certain non-caries outcomes [58]. Additionally, stabilized SnF_2_ pastes may provide periodontal and erosion benefits [58].

The main difference between OTC and professional pastes is the concentration and the amount of fluoride. OTC pastes typically contain 1000–1500 ppm fluoride and the 2019 Cochrane review supports these concentrations for primary and permanent dentitions, with larger absolute benefits at higher baseline caries risk—consistent with fluoride acting as an interfacial catalyst rather than a sterilant [5]. Professional dentifrices deliver ~5000 ppm fluoride, needed when daily acid challenges and specific substrates, such as exposed root/dentin or hyposalivation, high DMFT, orthodontic treatment, or recurrent caries, demand a higher interfacial fluoride activity to keep the system above the tissue-specific critical pH [59]. This explains why 5000 ppm delivers clearer advantages for root-surface disease and in xerostomic or high-risk scenarios, while standard OTC concentrations suffice for many low-risk individuals [5]. Randomized trials and syntheses in older adults show that 5000-ppm pastes arrest and maintain inactivity of root-caries lesions more consistently and reduce the likelihood of lesion reactivation up to two years [60]. Earlier meta-analyses already indicated that high-fluoride dentifrice is effective for non-invasive management of root caries; recent guideline summaries from professional bodies align with these findings [61]. In orthodontic patients, high-fluoride toothpaste can reduce or arrest white-spot lesions (WSLs), in some studies outperforming mouth-rinse regimens; nevertheless, varnish remains the most potent professional adjunct for WSL prevention, underscoring that delivery frequency and bioavailability determine outcomes [62].

Practical concentration-based indications and age-specific dosing recommendations for fluoride dentifrices and professional topical agents are summarized in Table 1 [5,6,63].

## 7. Effects of Fluoride on the Oral Microbiome

Fluoride alters the ecology and metabolism of dental biofilms in ways that complement its physicochemical effects on enamel. At the cellular level, undissociated HF permeates bacterial membranes and dissociates intracellularly, lowering cytoplasmic pH. Fluoride also binds Mg^2+^-dependent glycolytic enolase and inhibits the membrane F-ATPase proton pump. These actions suppress carbohydrate catabolism, acidogenesis, and acid tolerance which are key virulence traits of cariogenic organisms such as Streptococcus mutans. Classic enzymology established competitive inhibition of oral–bacterial enolases at micromolar fluoride, and newer reviews reaffirm this dual hit on glycolysis and proton extrusion [64].

Community-level consequences of these molecular effects have been demonstrated in vivo and in situ. A controlled mouse study that modeled human exposures (water levels and dental products) showed selective depletion of acidogenic taxa in the oral microbiome, with no comparable shift in the gut which is consistent with fluoride’s local bioavailability and upper GI absorption. Shotgun metagenomics indicated reduced representation of energy-harvesting pathways after fluoride, aligning with the predicted suppression of central carbon metabolism [65]. In humans, longitudinal multi-omics work revealed that three months of twice-daily brushing with 1450-ppm fluoride toothpaste shifted both the composition and activity of dental plaque in caries-active and caries-free adults; diversity was preserved while functional profiles moved toward a health-associated state. Subsequent addition of arginine further amplified alkali-generating pathways, but the fluoride-only phase already produced measurable ecological change [66].

Pediatric data support an ecological shift consistent with cariostasis. In a longitudinal cohort of preschool children sampled before and after fluoride varnish, investigators observed reduced abundance of cariogenic taxa with concurrent enrichment of health-associated genera and predicted changes in carbohydrate metabolism pathways, consistent with a transition from dysbiosis toward eubiosis following topical exposure [67]. Converging experimental and clinical microecology studies likewise indicate that fluoride suppresses growth and virulence traits of caries-associated organisms while favoring commensals linked to a more stable plaque ecosystem [68]. Importantly, these shifts do not entail wholesale loss of richness or collapse of community structure when fluoride is used at conventional preventive levels (e.g., dentifrices, varnish). Available evidence further indicates that topical applications modulate the oral microbiome in a caries-protective manner, whereas disruption attributable to community water fluoridation at recommended concentrations has not been demonstrated. Findings from animal models using higher systemic doses are not directly generalizable to typical human exposure [69]. This distinction between topical, interfacial biofilm effects and systemic ingestion is essential for interpreting microbiome data in public-health contexts.

Two considerations argue against a solely antimicrobial framing. First, fluoride primarily attenuates acidogenic and aciduric traits rather than sterilizing biofilms, consistent with the ecological plaque hypothesis and with preserved diversity alongside functional rebalancing [65]. Second, tolerance can emerge in vitro: cross-kingdom biofilm models show fluoride-resistant Streptococcus mutans supporting Candida albicans and maintaining EPS-rich, acidogenic biofilms despite sodium fluoride, blunting the anti-caries effect; how often and how stably this occurs in vivo under normal salivary flow and pH remains uncertain [70].

Taken together, the evidence points to a consistent pattern. At the molecular level, fluoride lowers bacterial acidogenesis and acid tolerance by inhibiting enolase and F-ATPase and by acidifying the cytoplasm. At the community level, routine topical exposures shift plaque function and composition toward a less cariogenic state without reducing overall diversity. Available studies in humans do not indicate adverse oral or gut microbiome effects at recommended preventive concentrations; however, this evidence base remains limited, and findings for higher systemic exposures require cautious interpretation and further research [71].

Looking forward, priorities include: standardized, exposure-resolved microbiome endpoints in clinical trials of fluoride delivery modes; integration of metatranscriptomics and metabolomics to quantify real-time acid–alkali flux under fluoride; and surveillance for fluoride-tolerance phenotypes within clinical biofilms. These advances would sharpen causal inference and guide precision prevention strategies that leverage fluoride’s biofilm-modulating strengths while guarding against unintended ecological shifts.

## 8. Safety Considerations: Cytotoxic and Genotoxic Actions of Fluoride

The ability of fluoride to inhibit metalloproteins, by binding to an essential metal in its active site or by mimicking a substrate after forming a complex with metal [72], together with fluoride’s acidification and electrolyte homeostasis disruption, as well as cell organelle damage, summarizes the main mechanisms of fluoride toxicity [73]. Interpreting the toxicological profile of fluoride requires careful attention to exposure domain, since the dose, route, and duration of exposure differ markedly across systemic ingestion, high concentration experimental settings, and routine topical clinical use. Clinically meaningful risk, however, depends on whether exposure is chronic and systemic or brief and topical, and whether concentrations exceed those encountered in routine preventive use.

**Established risks from chronic high systemic intake.** Systemic fluoride intake has well-known risks and side effects related to increases in daily dose and prolonged exposure time, appearing when the optimal fluoride water level of 0.7 mg/L is doubled to over 1.5 mg/L, resulting in dental and/or skeletal fluorosis [74,75,76]. Damage to the nucleus and endoplasmic reticulum of ameloblasts and osteoblasts [77] results in impaired structure of the enamel and bone lattice. The mechanism of fluoride action on these cell parts is primarily based on protein synthesis inhibition, which results in reduced secretion of enamel matrix proteins [78]. DNA fragmentation, cytoskeleton impairment, apoptosis, and endoplasmic reticulum stress are additional mechanisms of fluoride systemic toxicity [79,80]. Systemic fluoride intake in relation to neurodevelopmental toxicity anchors to studies on neuronal cell lines and animal models, which revealed decreased signal transmission, cytoskeleton damage, and myelin and microtubule damage [81,82,83] as mechanisms of impaired neuronal activity. For drinking-water fluoride at the level of 0.7 mg/L, there is no sufficient evidence of adverse effects on children’s IQ or adult cognition [84]. Endocrine toxicity of fluoride emerges from its effect on glucose homeostasis, through inhibition of glycolysis and ATP production leading to increased glucose uptake [85], and from thyroid hormone imbalance, through thyroid cell membrane disruption, endoplasmic reticulum stress, and oxidative stress attributed to high blood fluoride [86], leading to a decrease in thyroid hormones. Both of these endocrine disruptions, glucose and thyroid, result from prolonged intake of high-fluoride doses. It should be emphasized that these systemic effects are associated with prolonged intake of elevated fluoride doses; their extrapolation to populations exposed to optimally controlled fluoride levels is limited, and susceptibility may vary with age, renal function, nutritional status, and total cumulative exposure.

**Experimental in vitro toxicity at high concentrations**. In experimental in vitro settings, fluoride has measurable cytotoxicity to buccal, gingival, and periodontal ligament cells when administered concentrations are high [87,88], and these effects result from induced oxidative stress, perturbed DNA repair enzymes, and increased chromosomal aberrations. Across oral cell models, millimolar sodium fluoride (NaF) impairs mitochondrial function, protein synthesis, and proliferation. In classic studies, loss of viability is reported at ≥4 mmol/L (≈76 ppm F^−^) after brief exposures, with broader mitochondrial suppression as dose rises—levels that exceed salivary peaks after routine toothbrushing but may occur with undiluted contact from concentrated agents [88]. Such concentrations substantially exceed those attained intraorally during normal product use and are therefore informative regarding mechanism rather than directly predictive of clinical risk.

**Routine clinical topical use.** Evidence regarding routine clinical topical use is more reassuring, but it should be interpreted within the limits of the available data. In humans, standard home-use regimens with fluoride toothpaste or mouthrinse in preventive ranges (approximately 450–1450 ppm F), used twice daily, have not been associated with cytogenetic or genotoxic adverse effects in buccal mucosal cells under the studied conditions [89,90]. Clinically relevant concerns are therefore most plausible when concentrations and contact times substantially exceed normal preventive practice, emphasizing the importance of matching product strength to caries risk and minimizing unnecessary soft-tissue contact with concentrated agents.

## 9. Future Directions

Future studies should move beyond simple exposed vs. unexposed comparisons and measure how fluoride levels in plaque fluid rise and fall during everyday eating and drinking. Those measurements should then be tied to whether lesions arrest or reactivate in clearly defined groups, people with xerostomia, frequent snackers, or patients with orthodontic white-spot lesions. Building these data into trials would let us set doses by time spent in supersaturation rather than by headline concentration alone. Next, nanoscale analytics that map the ultrathin, partially fluoridated surface shell of enamel should be coupled prospectively with clinical endpoints to establish how pretreatments and contact times translate into durable reductions in enamel solubility in vivo. This is essential for rationalizing agent selection and pretreatment algorithms. For restorative materials, claims of superiority should rest on standardized secondary-caries outcomes at margins, with prespecified stratification by caries risk and salivary flow, concurrent reporting of plaque-fluoride at the tooth–material interface, and transparent recharge protocols. Finally, benefit–risk stewardship calls for biomarker-anchored cohorts that integrate total intake, adherence, and realistic soft-tissue exposures so that safety signals align with clinical practice.

A further frontier concerns the diagnostic implications of an interface-confined mechanism. If fluoride preferentially hardens the outer enamel while subsurface or dentinal demineralization can continue, conventional radiographic thresholds—which infer lesion depth from radiolucency as a proxy for mineral loss—may underestimate activity, and a lesion that appears radiographically stable over time is not necessarily arrested rather than progressing below the detection threshold. This concern is reinforced by the limited sensitivity of bitewing radiography for dentinal lesions in such presentations; in vitro comparisons against histological gold standards report radiographic sensitivity for dentin-extending lesions as low as 0.09, far below visual classification systems [91]. In parallel, the rapid emergence of artificial-intelligence-assisted caries detection introduces a specific caveat: convolutional neural networks trained on bitewing radiographs have shown promising performance for early interproximal lesion detection, yet their output depends on the grayscale gradients on which they are calibrated [92]. Where fluoride alters the expected mineral-density profile across the dentino-enamel junction, algorithms trained on standard lesion morphologies could generate false negatives, underscoring the need for fluoride-aware training datasets and prospective validation before automated radiographic screening is widely adopted [93,94].

## 10. Conclusions

Fluoride’s cariostatic value rests on robust, convergent evidence. It acts predominantly at the tooth–biofilm interface—through adsorption, ultra-shallow lattice fluoridation, and pH-responsive surface reservoirs rather than wholesale conversion to fluorapatite—where frequent, low-level exposures suppress demineralization and accelerate remineralization during acid challenges. Clinically, twice-daily use of appropriately dosed fluoride dentifrices remains the foundation of caries prevention, with professional modalities and high-fluoride formulations adding measurable benefit in defined high-risk contexts such as root caries and xerostomia. These adjuncts complement, but do not substitute for, meticulous biofilm control and sound adhesive dentistry.

## Figures and Tables

**Table 1:** Fluoride products: concentration, age-specific dosing, and clinical indications.

Product Category	Fluoride Concentration	Primary Indication	Key Patient Groups/Examples	Practical Notes
OTC fluoride toothpaste	1000–1500 ppm F	Routine caries prevention	Most children, adolescents, adults	Brush twice daily; advise minimal rinsing to prolong fluoride contact time [5,63]
Pediatric OTC dosing	1000–1500 ppm F	Safe/effective early-childhood use	<3 years; 3–6 years	<3 years: smear/rice-sized; 3–6 years: pea-sized; adult supervision; minimize swallowing [6,63]
Prescription high-fluoride toothpaste	~5000 ppm F (1.1% NaF)	Enhanced control of active/recurrent disease	High-risk patients ≥6 years (root caries, xerostomia, high DMFT, recurrent caries; selected orthodontic WSL scenarios)	Use under professional guidance; monitor lesion activity and integrate salivary + behavioral management [5,63]
Professional topical fluoride (varnish/gel)	High concentration (varnish/gel formulations)	Risk-based adjunct for lesion control and prevention	High caries risk; non-cavitated lesions/WSLs; root caries risk; patients with limited self-care capacity	Emphasize technique and risk-based frequency; avoid prolonged soft-tissue contact; integrate with daily fluoride exposure [5,6,63]

Abbreviations: OTC, over-the-counter; NaF, sodium fluoride; WSLs, white-spot lesions; DMFT, decayed-missing-filled teeth.

## Data Availability

No new data were created or analyzed in this study. Data sharing is not applicable to this article.
